# Exploring local knowledge and perceptions on zoonoses among pastoralists in northern and eastern Tanzania

**DOI:** 10.1371/journal.pntd.0005345

**Published:** 2017-02-01

**Authors:** Peter Ernest Mangesho, Moses Ole Neselle, Esron D. Karimuribo, James E. Mlangwa, Kevin Queenan, Leonard E. G. Mboera, Jonathan Rushton, Richard Kock, Barbara Häsler, Angwara Kiwara, Mark Rweyemamu

**Affiliations:** 1 Department of Veterinary Medicine and Public Health, Sokoine University of Agriculture, Morogoro, Tanzania; 2 Southern African Centre for Infectious Disease Surveillance, Sokoine University of Agriculture, Morogoro, Tanzania; 3 Department of Health Systems and Policy Research, National Institute for Medical Research, Amani Research Center, Muheza, Tanzania; 4 Department of Pathobiology and Population Sciences, Royal Veterinary College, University of London, London, United Kingdom; 5 Directorate of Information Technologies and Communication, National Institute for Medical Research, Dar es Salaam, Tanzania; 6 Department of Development Studies, Muhimbili University of Health and Allied Sciences, Dar es Salaam, Tanzania; Texas A&M University College Station, UNITED STATES

## Abstract

**Background:**

Zoonoses account for the most commonly reported emerging and re-emerging infectious diseases in Sub-Saharan Africa. However, there is limited knowledge on how pastoral communities perceive zoonoses in relation to their livelihoods, culture and their wider ecology. This study was carried out to explore local knowledge and perceptions on zoonoses among pastoralists in Tanzania.

**Methodology and principal findings:**

This study involved pastoralists in Ngorongoro district in northern Tanzania and Kibaha and Bagamoyo districts in eastern Tanzania. Qualitative methods of focus group discussions, participatory epidemiology and interviews were used. A total of 223 people were involved in the study. Among the pastoralists, there was no specific term in their local language that describes zoonosis. Pastoralists from northern Tanzania possessed a higher understanding on the existence of a number of zoonoses than their eastern districts' counterparts. Understanding of zoonoses could be categorized into two broad groups: a local syndromic framework, whereby specific symptoms of a particular illness in humans concurred with symptoms in animals, and the biomedical framework, where a case definition is supported by diagnostic tests. Some pastoralists understand the possibility of some infections that could cross over to humans from animals but harm from these are generally tolerated and are not considered as threats. A number of social and cultural practices aimed at maintaining specific cultural functions including social cohesion and rites of passage involve animal products, which present zoonotic risk.

**Conclusions:**

These findings show how zoonoses are locally understood, and how epidemiology and biomedicine are shaping pastoralists perceptions to zoonoses. Evidence is needed to understand better the true burden and impact of zoonoses in these communities. More studies are needed that seek to clarify the common understanding of zoonoses that could be used to guide effective and locally relevant interventions. Such studies should consider in their approaches the pastoralists’ wider social, cultural and economic set up.

## Author summary

Zoonoses are diseases transmissible between animals and humans. Risk factors include animal slaughter, the handling and preparing food of animal origin and particularly the consumption of such food when raw or undercooked. Pastoralists are daily in contact with their livestock and are likely to be more frequently exposed to zoonotic pathogens. However, very few studies have focused on the understanding of zoonoses and related risk factors among pastoralists of Tanzania. This was striking considering pastoralists’ close bond with and reliance on livestock for their nutritional needs and livelihoods. Our study was implemented in pastoral communities located in two different ecological zones of Tanzania. In all ten communities visited no local term for zoonoses existed, yet participants recognised there are some symptoms that appear in both animals and humans. However, these were not thought to be harmful to humans and people did not perceive the animal products from which they originate to be dangerous. Other zoonoses were known because community members received a diagnosis from a health facility. We also learnt that cultural practices relating to the preparation and consumption of food of animal origin were considered more important to the community than the risk of infection. The findings of this study highlight the gaps in knowledge of zoonoses among pastoralists in Tanzania and the importance of social and cultural practices. Therefore, we propose that interventions to control zoonoses should be informed by research of peoples’ behaviours related to handling of animals and consumption of animal source foods.

## Introduction

Zoonoses account for the most commonly reported emerging and re-emerging infectious diseases (EIDs) in Sub-Saharan Africa [1]. There is a large number of human diseases that originate from both domestic and wild animals (900 +), and a smaller number of zoonoses (250+), which are diseases transmitted between animals and humans [2]. The frequency of occurrence of zoonoses is relatively small in the overall burden of human disease but zoonotic EIDs are of major concern globally [3]. Although zoonoses have been reported in Africa, the continent is the least capacitated in terms of technological advancement and capacity for the early detection and control of infectious diseases in both human and animal populations [4–6].

Control of zoonoses is beneficial for developing economies and human health, as it reduces morbidity and mortality, saves costs for disease management and increases productivity, health and well-being [7]. Livestock keepers in Africa in general and Tanzania in particular, face a potential double burden of animal and human diseases as they fend for their livelihoods. They are a group at risk of zoonosis due to livelihood and livestock keeping practices, which puts them in direct contact with their livestock and wildlife and therefore at high transmission risk [8–11]. Another important transmission pathway is related to the consumption of animal source foods, in particular specific local practices like consumption of raw or undercooked meat, and drinking raw blood/or milk. The practice of sharing community water sources with livestock constitutes another potential source of zoonotic infection [12,13]. In Tanzania, a number of studies have documented the presence of infections that are potentially zoonotic. They include bovine tuberculosis [14,15], rabies [16,17], brucellosis [18,19], anthrax [20,21]and Rift Valley fever (RVF) [22–24]. Most of these diseases affect people’s capacity to effectively manage their livestock and abate food insecurity.

Pastoralists have lived close to their animals for millennia and possess a wide repertoire of local traditional knowledge systems in the identification and addressing of both human [25] and animal afflictions [26]. Some scholars have gone as far as comparing pastoralists diagnostic skills to that of the modern medicine [27]. Thus, early detection and response to illnesses are important steps towards effective interventions. Such knowledge is important to facilitate communication between pastoralists on the one hand, and animal and human health experts on the other [28,29]. In spite of the socio-political changes affecting pastoralists in sub-Saharan Africa, they still practice a traditional way of life that is rooted in a cultural system, which guides all spheres of life, including human health. Health and illnesses are approached both using naturalistic and ritual activities which inform their causes and the subsequent interventions. The Maasai pastoralists, like many other African traditional societies are said not to distinguish between religious beliefs and empirical knowledge when it comes to seek healing [25,30] but prescription of the most relevant treatment has to come from understanding the source of the illness since they make a distinction between natural and supernatural caused illnesses.

For many illnesses pastoralists rely on a number of options, which include local healers, spiritual diviners, midwives and modern medical care. Illnesses that are believed to naturally occur are addressed using medicinal plants and/or modern medical care, and those that are believed to arise from misfortune or unusual causes are addressed through consulting a traditional diviner [30]. The breadth of pastoralists’ knowledge and usage of medicinal plants demonstrates the extent of treatment, which relies on local knowledge [31]. For example, Fratkins’ account of the Samburu pastoralists in northern Kenya lists more than 90 medicinal plants that are believed to treat more than 50 different ailments and conditions [25]. Similarly, a number of scholarly works have documented medicinal plants and corresponding treatment for the Maasai of Eastern Africa [31,32], Dinka of Sudan [33] Fulbe and Arabs pastoralist groups of Northern Cameroon [29] and pastoralists from Ethiopia [34]. Some of the ailments covered include fever, malaria, chest congestion, wounds, burns, women's stomach pain, hepatitis, snakebites, stomach problems, colds, polio, gonorrhea, arthritis and abortions among others. There are also additional human afflictions and misfortunes that are believed to come from not following relevant culturally informed diets, a process that is said to ‘pollute’ the body and block internal circulations. For example, the consumption of wild animals is prohibited among the Maasai [25].

Qualitative studies on zoonoses, which attempt to assess knowledge and perceptions of zoonoses and risky behavioural practices towards transmission in Tanzania are few and are still rather descriptive [28,35] There have been a few studies on knowledge, attitudes and practices among pastoralists, but their designs have important limitations [36]. For example, Shirima and colleagues [8] noted that Maasai pastoralists named malaria, east coast fever (ECF), mastitis, allergies, typhoid fever and cancer as zoonotic. The authors suggested that pastoralists fail to correctly recognize the animal sources of certain infections. Therefore, there is a need for well-planned qualitative analytical studies among pastoralists. Such studies are likely to support the integration of traditional knowledge systems into modern health approaches that can improve zoonosis management. This study was carried out to understand perceptions and knowledge on zoonoses among pastoralists in Ngorongoro, Kibaha and Bagamoyo districts of Tanzania. Understanding people’s perceptions about zoonoses and other infectious diseases, within their social and cultural context is important in order to provide relevant evidence for planning appropriate interventions [37].

## Material and methods

### Ethics statement

The Medical Research Coordinating Committee of the National Institute for Medical research approved the study (Ref. NIMR/HQ/R.8a/Vol. IX/1649). Prior to all interviews and discussions, information about the study, which included purpose of the research, confidentiality and uses of data, was read out to the participants and a local translator translated into the local language of the participant where the National Kiswahili language was not popular. Verbal informed consents to participate and allow recording of the interviews were obtained from all participants before beginning.

### Introduction and study sites

**Fig 1:**
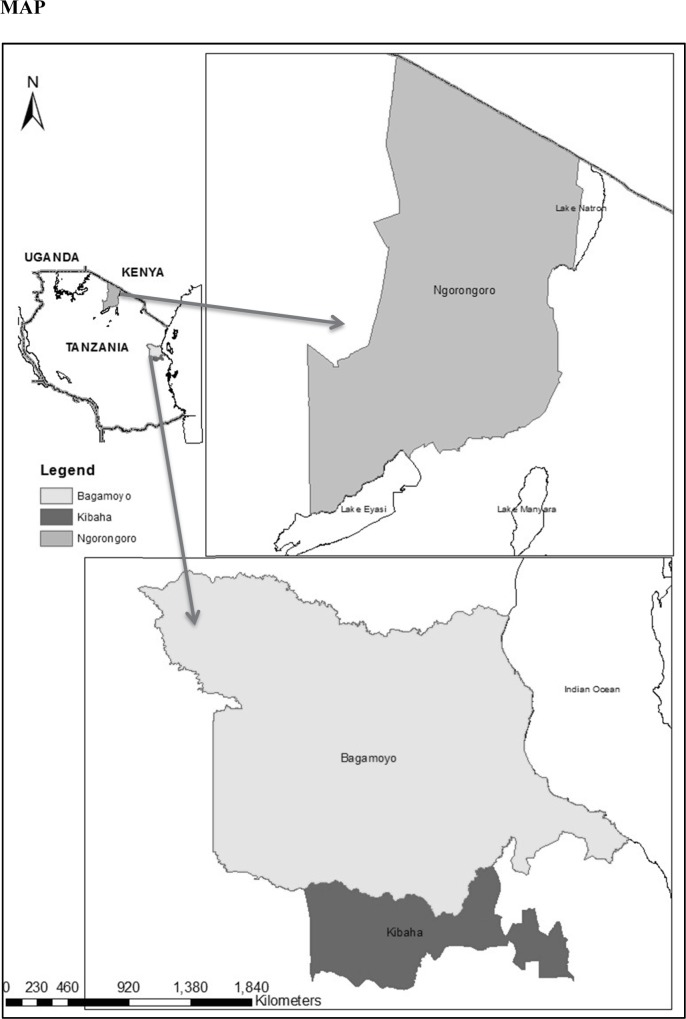
Study sites map.

The study was carried out as part of a larger programme on infectious disease control among Maasai pastoralists of northern and eastern Tanzania with the aim of formulating participatory disease control interventions through research. This research was implemented between May 2014 and May 2015, in Ngorongoro (Northern Tanzania), and Kibaha and Bagamoyo (Eastern Tanzania) districts (Fig 1). A total of ten (Ngorongoro = 6; Kibaha/Bagamoyo = 4) villages were included (Table 1).

**Table 1:** Study sites and major pastoral ethnic groups.

District	Division	Village	Ethnic Group[s]
Ngorongoro	Ngorongoro (Ngorongoro Conservation Authority)	Osinoni	Maasai
		Olbalbal	Maasai
		Nainokanoka	Maasai
	Loliondo	Enguserosambu	Maasai
		Mageri	Batemi/Sonjo
	Sale	Malambo	Maasai
Kibaha	Magindu	Magindu	Maasai
		Miziguni	Maasai
	Kwala	Kwala	Sukuma and Datooga
Bagamoyo	Pera	Chamakweza	Maasai

The study villages were therefore selected from these three program districts. The selection of the villages for this study was purposefully done by taking into account the contrasting landscape within each district. These included the farming system practiced [pastoralism or pastoralism and crop cultivation] altitude, and location [located within a conservation or not] and to a lesser extent, ethnic groups.

The Ngorongoro District is comprised of arid and semi-arid lands, more favorable to nomadic livestock keeping than crop agriculture [38]. Ngorongoro District is home to a number of pastoral communities, typical of sub- Saharan Africa, where traditional transhumance pastoralism, wildlife and crop production exist side by side. The district is inhabited mainly by the Maasai, a semi-nomadic ethnic group, who make up approximately 85% of the population [39]. The district is also divided into areas determined by two main socio-economic activities. The Ngorongoro Conservation Area Authority (NCAA) in Ngorongoro Division, where pastoralists reside with wildlife but cultivation is strictly disallowed by regulation. Pastoralists in the NCAA receive subsidized maize parcels once in a while to compensate for non- cultivation. Beyond the NCAA, in the Loliondo and Sale divisions to the north, a combination of wild life game controlled areas and cultivation exist side by side.

Kibaha and Bagamoyo districts are humid savannah peri-urban sites situated along the eastern coastal hills with limited human-wildlife-domestic animal interactions. The sites comprised mainly of Maasai pastoralists who share their borders with crop farmers. Other pastoral groups such as the Sukuma and Datooga have recently migrated to Kibaha district in search of pasture, water and markets [40,41]. In our study Sukuma pastoralists were included as participants from Kwala division as they are the largest and settled pastoralist group in the area compared to the Datooga and Maasai. While our main focus was on Kibaha pastoralists, the inclusion of a village situated in Bagamoyo district was necessary due, not only to the shared boundaries with neighbouring pastoralists in Kibaha but also shared resources such as pasture, water and markets.

### Data collection

Multiple data collection techniques were used to gather data for this study. Focus Group discussions (FGDs) were mainly used, which were combined with interviews and observations, and participatory epidemiology. The observations were combined with interviews and were conducted while participating in events of interest in the community [42]. These events included attending local markets, slaughter of animals, grazing and herding livestock, milking cattle and identification of livestock diseases. In addition, participatory epidemiology was employed through asking group participants to rank and weigh illnesses in the order of importance. During discussions communities were involved in defining and prioritizing veterinary-related problems and solutions [43].

All data collection activities were conducted by trained researchers supported by a translator who was conversant in the participants’ local language and the national, Kiswahili language, which was used by the facilitators and the interviewers. One of the research team member (MN) is a Maasai and spoke the Maa language fluently. He assisted in most of the interviews and FGDs. Some participants, especially the Maasai from the coastal districts of Kibaha and Bagamoyo, spoke Kiswahili more fluently compared with participants from Ngorongoro.

FGDs using a question guide were conducted in separate groups consisting of men or women. The guide included topics on human and animal health, health-seeking practices for humans and animals and livelihoods and food security. Most discussions lasted about one hour. In addition, interviews were done with village leaders, local chiefs, medical and local livestock officers, and district officials. Some of these were informal while others were prearranged [42]. The informal interviews did not necessarily last long as they were conducted on the spot to clarify aspects of the study observed in the community or during the FGDs. Furthermore, in-depth interviews were conducted with livestock keepers during dry season migrations, market days, at dipping facility sites or at local slaughter slabs.

Both in-depth interviews (IDIs) and FGDs were conducted in a flexible and iterative manner that adapted to the topics emerging during data collection. As new themes emerged and were considered important, additional questions were formulated for follow up probes with other interviewees. For example, it became apparent to the team that to better understand how people perceive the potentiality of zoonoses, it was necessary to conduct observations and interviews at the point of meat inspection.

Ranking of important perceived zoonotic illnesses were conducted by first asking to list the commonly occurring perceived illnesses and then proceed to rank based on their discussions. A simple ordinal scheme was used whereby the illness perceived by the participants as having greatest impact on livelihoods and affecting their daily engagement in production activities was assigned a value of 1, the illness seen as the second got a value of 2, and so on. During the ranking process it was important to obtain a common agreement of the common illnesses and their perceived status. To do so, participants in groups were encouraged to discuss among themselves and agree on whatever they thought were the major syndromes encountered in their village.

### Data analysis

Discussions from the digital recorders were transcribed aided by the F4 transcription software. The transcripts that were in Kiswahili were translated to English for analysis by the research team. The Maa speaking researcher, directly translated transcripts that were in Maa, into English. The results obtained from each illness were analyzed guided by the content analysis deductive method [44] based on a categorization matrix. Data was coded according to the categories preferred under corresponding main themes. Initial analysis commenced while still in the field through expanding the notes after the interviews and FGDs were conducted [42]. This also included cross-checking the described symptoms with human and animal health professionals in the field. Coding initially was done using Nvivo10 (QSR International) by identifying themes and sub themes related to the topic of interest, such as the perceptions and local understanding of the meanings of zoonosis. The transcripts were read several times to get an overall understanding of the messages in the text and the codes were refined, added or removed depending on their significance to the developing data. Hand written notes from numerous follow-up short and long interviews were manually incorporated into the related themes. In each theme and sub theme of interest, discussions were built around them and links between were identified from which the main conclusions were reached. Illness rankings were analysed using a simple descriptive ranking with the illness receiving the highest rank toping the list followed by the rest.

## Results

Demographic data of participants are summarised in Tables 2 and 3. Overall a total of 203 individuals (females = 110, males = 93) participated in the discussions and the illness ranking exercises. Of the participants, 61 and 142 people represented Kibaha/Bagamoyo and Ngorongoro districts, respectively. Most of the participants were married (88%). About half (45.8%) of the respondents had no formal education; 49.8% had completed primary education while 4.5% had above primary education. In addition, a total of 20 people were participated in face-to-face interviews (Table 4). Participants owned different types of animals, which in the order of importance included cattle, goats, sheep, donkeys, dogs, chickens and cats. However, only participants from the coastal district sites owned chickens and cats. Summaries of the main thematic analytic results are presented on Table 5.

**Table 2:** Socio-demographic characteristics of the FGD participants.

		Kibaha/Bagamoyo		Ngorongoro		Total	
Category	Sub Category	Number	Percent	Number	Percent	Number	%
**Sex**	Female	33	54.1	77	54.2	110	54.2
	Male	28	45.9	65	45.8	93	45.8
**Total**		**61**	**100.0**	**142**	**100.0**	**203**	**100.0**
**Age in years**	18–40	30	49.2	75	52.8	105	51.7
	41–80	31	50.8	67	47.2	98	48.3
**Total**		**61**	**100.0**	**142**	**100.0**	**203**	**100.0**
**Marital status**	Single	1	1.6	7	4.9	8	3.9
	Married	47	77.0	132	93.0	179	88.2
	Widowed	12	19.7	3	2.1	15	7.4
	Divorced	1	1.6	0	0.0	1	0.5
**Total**		**61**	**100.0**	**142**	**100.0**	**203**	**100.0**
**Education level**	None	33	54.1	60	42.3	93	45.8
	Primary	23	37.7	78	54.9	101	49.8
	Secondary	4	6.6	2	1.4	6	3.0
	AboveSecondary	1	1.6	2	1.4	3	1.5
**Total**		**61**	**100.0**	**142**	**100.0**	**203**	**100.0**

**Table 3:** FGDs per village, district, division and ethnic groups.

District	Division	Village	Number of FGDs	Major ethnic Group
Ngorongoro	Ngorongoro (Ngorongoro Conservation Authority)	Osinoni	4	Maasai
		Olbalbal	4	Maasai
		Nainokanoka	4	Maasai
	Loliondo	Enguserosambu	3	Maasai
		Mageri	4	Batemi/Sonjo
	Sale	Malambo	4	Maasai
Kibaha	Magindu	Magindu	2	Maasai
		Miziguni	2	Maasai
	Kwala	Kwala	2	Sukuma & Datooga
Bagamoyo	Pera	Chamakweza	2	Maasai

**Table 4:** Key informant interviews.

District	Title	Gender	Number
Ngorongoro	Village Elder	M	3
	Veterinary officer	M	1
	Human health officer	M/F	2
	Field Livestock officer	M/M/F	3
	Pastoralist leader	M	2
	Women elder	F	1
Kibaha	Village Elder	M	1
	Pastoral Leader	M	2
	Veterinary officer	M	1
	Human health officer	M	1
Bagamoyo	Village elder	M	1
	Pastoral leader	M	1
	Livestock officer	M	1
Total			20

**Table 5:** Main thematic analysis results summaries.

Focus	Main Themes	Sub-themes
Disease cross infections	The notion of zoonosis	Existence and/or non-existence of zoonosis
		Infected animal by products as harmful to humans
		Comparable syndromes in animals and humans
		Knowledge of infections to humans from health facility diagnosis
Perceived pathways of zoonosis via animal by products	Consumption of animal products	Seasonal resource search migrations
		Age set initiations including circumcision
	Social and cultural responsibilities	
		Social gatherings: for example weddings; and the notion of good food
	Notions of fresh blood and animal internal organs	
		Women needs following birth of a new born child
	Sense of closeness with animals	
		Raw milk and undercooked meat debates
		Physical contact with animal or animal by products
Regulation of pastoralist consumption patterns	Pastoralist as non-conformist to rules guiding meat use	Pastoralists understanding of infection from animal products
		Livestock inspection and the interest in revenues
	Officials interfering with pastoral way of life	The importance of fulfilling social and cultural obligations vs rules guiding animal handling

### Understanding the term ‘zoonosis’

To understand how participants perceived zoonosis, it was important to first focus on the etymology of the terms of illness used in the local language. Since the term “zoonosis” did not exist in the local language as a single word, posing the question demanded a careful but broad interpretation of the term. In initial discussions, the study participants mentioned two perceived illnesses that they categorized as ‘zoonoses’. Particularly with participants of Kibaha and Bagamoyo districts the idea of the existence of ‘zoonoses’, especially from infections as a result of consuming meat/milk/blood from a sick animal, was met with rather a long pause and counter questions as to whether there is such a possibility of humans getting infection from afflictions that affect their livestock. Some questioned repeatedly if humans can get infected from consuming meat. In all groups, it was notable that participants mentioned fewer syndromes transmitted from livestock to humans as compared to naming illnesses and syndromes that affected their livestock.

There was a sharp contrast in the way pastoralist groups from the eastern and northern districts talked about illnesses that are potentially zoonotic. In Ngorongoro District some participants recognized that some illnesses were shared between livestock and humans, although there were also individuals who were skeptical. On the other hand, participants from coastal districts were generally skeptical about the existence of zoonoses. Zoonotic illnesses were generally not perceived as a threat or harmful among those who said they were aware of infections contracted from animals. In the coastal districts however, the notion of zoonosis was reported as uncommon. In some groups people were made aware of this possibility for the first time through our discussions. Some of the responses below attest to this observation:

*“We eat meat*, *we drink milk*, *we eat and drink the fat from the animals*, *every meat*, *but we do not get any illness because of that*.*”* (FGD-01-Female 4- Magindu village-Kibaha)

*“We do not believe there are illnesses in the milk because we have been milking these animals for a very long time*. *I have grown up from drinking this milk until today; why are you telling me they are bad?”* (FGD-01-Male– 3 Chamakweza village–Bagamoyo)

Although some participants reported to have heard of zoonotic illnesses such as tuberculosis and anthrax, this was not well received by the rest of the discussants who refuted such a possibility. In the male FGD at Magindu, a long debate among the participants took place following the moderator’s posing of the question about the presence of zoonosis. One member responded to the existence of *emboroto* (mostly referring to anthrax by most pastoralists in northern Tanzania) in the community. Signs mentioned included swellings on some parts of human body. One middle- aged participant insisted it came from eating meat but most of the members seemed to disagree. Although they knew the syndrome, they had never heard that it was acquired from consuming meat or milk:

*“We were discussing that because what we are hearing is something new*, *which we have just discovered here*. *We did not know that but what we know when someone has those swellings we call those* emboroto. *May have swellings on the leg*, *or somewhere else*. *But that this particular swelling is either from livestock to humans*, *this difference we never knew*. *Also that you could get a swelling from eating animal products is something we did not know*. *This is very new to us and that is why we are asking each other here*, *if this* emboroto *we get sick from is also the same in cattle*.*”* (FGD– 01-Male 6- Mizuguni- Kibaha)

The discussion from this group showed that there was unclear understanding on the source of this illness and they thought to be something else, as anthrax has not been reported in the community before.

The discussion guide therefore had to be expanded with a number of follow up questions and probes. For example, we asked what animal diseases or health problems they think and believe they could acquire or become infected with. Another follow-up question was what [animal] syndromes they can acquire from staying close or living with their animals. Further, in many instances participants were asked what syndromes they can acquire from consuming animal products such as meat, blood or milk. These questions resulted in broad responses and more discussions, which were guided more by the participant’s perspectives. This approach allowed participants to be as detailed as possible as to what they could group as ‘zoonotic’ and this led to an interesting listing of perceived zoonoses (Table 6).

**Table 6:** Perceived zoonoses as ranked by all groups.

Rank	Ngorongoro District		Kibaha and Bagamoyo Districts	
	English	Local term	English	Local term
1	Brucellosis	Brusela	Tuberculosis [TB]	TB
2	Foot and mouth disease	Oloirobi	Anthrax	Emboroto
3	Anthrax	Emboroto	Rabies	Rebis
4	Rabies	Rebis	Skin fungal infections	Ndororo
5	Black quarter	Emburuo	Respiratory infections	Mapafu ya kikohozi cha mbuzi

### How pastoralists described syndromes attributed to zoonoses

Throughout the discussions and interviews, participants differentiated zoonoses from two main angles. These were illnesses that they believed to have been around for many years; and illnesses handled at a health facility and from receiving a diagnosis. For the latter, which included tuberculosis and brucellosis, they were informed by health workers at health facilities that the infection is acquired from consuming animal products such as milk, meat and blood from infected animal. The participants in the FGDs referred to these illnesses as ‘hospital diseases’. The group of syndromes that have always been there included anthrax (known as *emboroto*) and foot and mouth disease (known as *oloirobi* in the Maa language).

Syndromes in humans attributed to zoonoses were identified by correlating syndromes as they presented in suspected animals. For example, the correlating syndromes in humans for *oloirobi* were blisters around the mouth, flu, nasal discharges, sneezing, fever, cough and diarrhea. Participants in one group reported that the syndrome (*oloirobi*) could also affect pregnant women, in which the illness could be transmitted to the unborn child and also from one human to another through the air. The syndrome was described to be self-limiting with spontaneous recovery without treatment. Some people would use analgesics such as paracetamol to relieve the associated pain. Furthermore, the illness occurrence in human is seasonal and coincides with the occurrence of foot and mouth symptoms in cattle. It was perceived to come from consumption of milk during the peak of rainy season (April-July) when milk is plenty. However, many participants in the Ngorongoro believed this seasonal occurrence of the illnesses has also changed and now it may occur at any period during the year (appearing at least three times).

Anthrax (*emboroto*) was perceived locally to have been present for generations. Livestock keepers reported that mostly signs were only recognized after the animal had died although some were apparently health before. The syndrome is characterized by swellings, and oozing of blood from the nostrils and/or ears. When dissected some parts of the carcass are noted to be black in colour. In humans, recognised symptoms include swellings, general body malaise, fatigue and loss of appetite. The swellings, later turn in to black spots during healing. In the local Maa language this black spot is called *emboroto*. It is also the blackness that is recognized in a suspected case in an animal, which is observed only after death.

On the other hand, ‘hospital diseases’ syndromes were said to be diseases that the health care providers will diagnose in people. Interestingly, in most of the groups in Ngorongoro, participants referred to *brusela* (brucellosis) as a new illness. Upon further probing the illness was considered new because they had only been told about it by health workers from year 2007, although a few cited it from the early 2000. In Osinoni (Ngorongoro) it was given a local name *nangidaeton*- meaning discomfort when moving, the feeling of general body malaise. But they insisted it was not there before 2007. This suggests that it was perceived as an emerging illness.

Among the participants in eastern Tanzania, tuberculosis (TB) was perceived as somewhat uncommon among pastoralists and its link with milk seen as untrue. For example, it received some resistance among the Magindu male groups for its presence among pastoralists. Many discussants reported that they only hear about the TB when a relative is taken to hospital and diagnosed with the disease to be told by health care providers that it could be because of drinking unpasteurized milk. Participants strongly disagreed that they were in danger from drinking milk and directed that notion to other social or cultural factors. Those who had heard of the possibility of milk being a source of TB did not directly relate the TB to the milk itself but to something else:

*“We hear people say that you can get TB from milk… people who get TB more often are the* Waswahili *than the Maasai although we are the ones who drink unpasturised milk more than them*.*”* (FGD-01-Male 7- Miziguni, village, Kibaha)

*“We pastoralists are told that we are more at risk [of TB]*. *We get it from cattle because we hear that cattle also get it*. *Also there is the hair from the animal*, *because we do not filter or boil the milk it affects us…the hair drops into the milk and if you drink the milk with hair they go in and block in the lungs*. *So everyday that we drink without filtering they add up and you end up getting the chest illnesses*.*”* (FGD-01-Male- 3- Magindu village, Kibaha)

Other perceptions of possible syndromes locally identified by the participants demand further scientific investigation if they actually exist or do fall within the category of zoonotic illnesses. For example *mapafu ya kikohozi ya mbuzi -*a syndrome that was said to attack only goats with prominent coughing symptoms was mentioned as an infection that later occurs in humans, manifesting with more or less similar symptoms. This syndrome is acquired possibly through inhalation when staying close to goats or breathing the same air with goats. Equally, a condition locally called *ndororo*, which was thought to be a fungal infection in humans was described to occur on the feet of people who walk barefoot in cattle slurry or step on raw livestock remains. However, these symptoms were considered not to be harmful to people’s health, as they could be managed with syrups and ointments from drug shops.

### Possible reasons for the discrepancy between Ngorongoro and the eastern districts

Interviews with Ngorongoro District officials and local health personnel revealed that there have been a number of health activities targeting zoonoses. In particular, project activities by a number of academic and research institutions have been taking place in recent years targeting zoonoses especially human brucellosis, through diagnosis and treatment. The health officer believed this has raised the awareness about the disease and others zoonoses among the people who seek medical care at their facilities. The capacity to conduct disease diagnosis in Tanzania is available at the district hospitals. The health officials for Ngorongoro district reported that to a large extent, interventions in terms of both community and hospital based clinical studies, whose results have been published [19,45], have facilitated and built local capacity to readily diagnose and treat brucellosis, which in turn has raised awareness in the community. In the process the awareness of other zoonoses was also raised since the experts do provide health education on the existence of other zoonoses and their pathways while focusing on brucellosis. On the other hand, all health facilities found in villages and neighbouring towns in Kibaha and Bagamoyo district, which are frequently used by the pastoralists from the area, reported not to have the capacity to perform tests for zoonoses such as brucellosis.

### Local knowledge around potential risks of infection acquired through meat consumption

Given that the understanding of the concept of a zoonosis was lacking by the pastoralists, it prompted the need to further understand their general perceptions of illnesses and the links to food sources. It was important to understand perceptions of livestock disease to shed light on how they might perceive zoonotic disease possibilities. In all group discussions in both sites and in individual interviews with elders, respondents claimed that they did not fear any disease that affected animals:

*“We have been living with these animals for many years and we slaughter them when they are about to die*. *Diseases affecting livestock cannot affect us humans*, *it is difficult to happen*.*”* (Interview-Elder–Chamakweza-Bagamoyo)

None of the locally known livestock health problems were perceived as seriously harmful to humans. A common practice in Maasai tradition for example is to consume the meat from dead or ill livestock. It is believed that no matter what killed the animal in the first place, that the affected animal cannot harm again, [in Maa: *meyha engeeya nabo iltung’anak aare]*. In other words, the effect of the poison or illness ends in the affected animal and will not go to the person who eats meat of that animal.

*“…yes*, *it is true*, *an animal can be killed by an illness or poison*, *it [what killed the animal] cannot come out because the animal is the one that was targeted and it is already dead*. *Not me*. *So it is safe now to eat that animal*.*”* (Interview- Male pastoralist, Ngorongoro)

This was emphasised when talking about anthrax in cattle. Although it was perceived as a feared illness by a good number of participants, reported practices related to cadavers intensifies the notion of the lack of fear towards meat from dead animals. A local livestock field officer reported to have seen cases of meat been sliced off for consumption from an animal believed to have died of anthrax.

*“There was a case of anthrax reported...*..*We gave clear instructions that the dead animal should be buried and we were going there to do so*. *When we arrived we found some body parts were sliced out and the meat taken*. *When we asked why*, *they refused to talk*. *But I was not surprised because they do it all the time*. *We trying to educate them and it is our hope they will understand*.*”* (Livestock Field Officer—Ngorongoro district)

Interviews with livestock keepers revealed that during the dry season migrations in the search of water and pasture in the Ngorongoro District, the consumption of meat from suspected anthrax infected cattle, knowingly or unknowingly, was common. They said that they would dissect the animal after observing that it has not completely decomposed and that they will only consume the meat of body parts that appear to be safe. One elder reported that they can curb the effects of anthrax by consuming medicinal herbs known locally as *emporokway ekop* by boiling its roots and drinking the juice. They believe it totally eliminates any possibility of contracting anthrax. Another approach, reported to limit effects of suspected anthrax, is to dip a finger in the blood of the animal and touch the finger of blood on the upper part of the mouth. This was normally done by an elder. The act symbolizes stopping the spread of the infection to other people who will consume the meat.

The lack of fear of infected livestock was not limited to the Maasai pastoralists alone in this study. Among the Sukuma pastoralists in Kwala, the researchers observed the consumption of meat from a dead cow confirmed to have contagious bovine pleuropneumonia [CBPP]. The meat was well roasted after it was butchered and then later consumed. Members from neighbouring *bomas* were invited to partake in the feast including the researchers. When asked about practices of consuming that particular animal and other infected animals they too expressed fearlessness of consuming meat from infected animals.

In contrast to the Maasai, the Batemi FGD participants reported that they are more careful with what to eat although they also asserted that they do not fear to consume meat from suspected animal illness and they thought that an illness transmitted from cattle to cattle can never be harmful to humans. They said they would not eat meat of a dead animal not knowing what killed it in the first place or if it was from a known anthrax case. They also reported that they would not eat body parts that have been affected, although the lack of post slaughter meat inspection services is hindering them to practice this behavior:

*“We know it is difficult to tell if the meat is good to eat or not but we cannot just eat a meat of animal we find dead*. *We ask the boy who was herding what happened to it*. *But it is still difficult to know sometimes*.” (FGD-01-Male-03-Mageri village, Ngorongoro)

While most of the discussions and interviews suggested fearlessness in the consumption of meat from known sick animals, it seems there was a limit to that fearlessness. Fear of eating a dead or sick animal was reported to come from two aspects. First, it may come from the severity of the sickness. It was stated that should an unknown health problem affect and decimate a large number of animals then the perception that it may be harmful or dangerous to humans is agreed. However, if the sickness kills only a few, then there is no fear in consuming the dead animal. To the pastoralists, the death caused unto only few animals suggests that the illness may not be harmful.

Secondly, some Maasai pastoralists reported that they would consider the degree of decomposition of a dead animal before considering it unfit for human consumption. Reporting their experiences, local meat inspectors said that it takes a lot of effort to convince most of the pastoralists that meat from dead or sick animals is not fit for human consumption and needs to be disposed off according to stipulated government regulations. A Maasai pastoralist will mostly agree that a carcass is unfit for consumption if it contains multiple abscess in the liver or ribs:

*“Until they see with their naked eyes that the body parts are completely decomposed is when they will agree not to consume and have them buried*.” (Local Meat Inspector–Mageri-Ngorongoro)

### Further local knowledge about local customs of consuming raw blood as potential pathway for transmitting zoonoses

The pastoralists interviewed reported consumption of raw blood for various purposes such as rites of passage. For the Maasai, they reported that there were different occasions when taking raw blood is practiced. Young initiates consume raw blood after returning from circumcision ceremony. The main belief behind this custom was that the blood replenishes nutrients lost during circumcision. Equally, raw blood is an important meal for women who have given birth and in particular those who are thought to have lost blood due to bleeding. Raw blood is usually mixed with milk and given to the woman. The mixture provides important nutrients in terms of protein and fat that they consider necessary for the body in terms of energy production. Internal organs that can be consumed raw include kidneys, intestines and omasum. However, the custom in the consumption of raw meat was also strongly associated with a common belief about what is fresh and not fresh and the interpretation of freshness considers the time passed since the death of the animal. Blood that is cooked or cooled loses its freshness with time. Freshness of the blood means that the ingredients in the blood are still alive and will be immediately useful to the body. To the Maasai blood is also used as a drink when milk is not available especially in rare occasions like during distant grazing by young warriors known as *morani*.

The local understanding behind the value of rawness of meat and other internal organs is equally applicable to local knowledge about taking raw milk, a practice well defended despite known possibilities of sources of infections such as TB. Raw milk was reported in all groups as the main staple of the *Maasai’s* diet. It was also mentioned as important among Batemi and Sukuma groups interviewed but not to the extent emphasized by the Maasai. Raw milk is also the main meal for young boys (*Nyangulo*) and warriors known as *Morani* and *Koriangas*, who partake in migratory seasonal transhumance in search for pasture. It is stored in long gourds and sometimes mixed with special tree herbs known as *olirin*, *oseki*, *osinoni* and *ormisigiyo* to preserve the aroma of the milk and prevent the milk from contamination:

*“These tree herbs are special because*, *together with the gourd can protect it from contamination and sometimes they can stay a long time without going bad because they are very clean*.*”* (Interview-Elder- Enguserosambu- Ngorongoro)

This was evident during fieldwork with migrating herders in search of pasture and their livestock around the border of NCAA with Serengeti National Park during dry season pasture search. Raw milk is believed to contain important nutrients necessary for survival in these harsh environments and during long trips in herding cattle. The fat in the milk is believed to keep the body warm, the benefits of which are welcomed during the cold seasons. Furthermore, raw milk is used to defeat hunger and believed to stay for a longer time in the stomach than when milk is boiled. Boiling milk is believed contribute to fat loss and making the milk soft. Practically, milk is easy to obtain and can be taken straight from the cow and is perceived as safer than when it is handled and stored in different containers.

### Experiences from livestock officers on meat inspection at markets and slaughter places

An additional angle to understanding perceptions people hold about zoonoses was obtained at the auction markets and slaughter places visited. On the spot interviews of cattle and goat owners, including butcher-men suggested that the requirement of meat inspection is, by and large, perceived as the imposition of authority, conforming to the rules and regulations surrounding meat inspection, rather than a supportive service to prevent harm, such as zoonosis, to the consumer. When the question was asked “Why do you think meat inspection is done?”, a respondent in Chamakweza in Kibaha said it was because the government wanted revenue. This sentiment was echoed at auction markets in Endulen, Wasso, and Loliondo in the Ngorongoro district. Complaints by local animal health officials on the unwillingness on the part of pastoralists for meat inspection before local ceremonies were very common across the sites.

*“They do not care about us*, *they disrespect us*. *They will never call you for a meat inspection*. *They do not believe that they can get infected from livestock diseases*. *You have to really fight with them sometimes*. *But if you persist they hate you and it can ruin your work*. *So we decide to leave them to do what they want*. *We are not equipped to pursue those who do not comply with regulations*.*”* (Livestock Field Officer- Ngorongoro)

The extent to the practice of ignoring official orders goes very far with some people who will consume meat from animals declared unfit for human consumption. Some customs were reported to be so spontaneous to the extent that seeking services of a meat inspector was unfeasible. For example, slaughtering a goat to visitors, meat feasts by the young *morani* (warriors) out in the bush and the distance from human settlements. From the discussions and informal interviews, it was obvious that the perceptions of possible infectious diseases or illnesses a person could acquire from consuming meat from infected animals as a threat were not tagged as something serious among the pastoralists. What was important were meanings attached to the practices themselves. An educated local elder and chief said the reason for *Maasai* not believing the threat of illnesses from animal products, was because meat and milk form their main diet, and in different times [especially during migratory grazing periods] are their only meals. This has been the case for many generations and it was unthinkable to believe that their main and only meal could be harmful to them:

“*Cattle are the only source of livelihoods for the Maasai*. *People eat*, *drink milk and blood at different times of the year*. *When the* morani *go out to herd animals that is their main meal*. *The meat from the Maasai animal is life*, *and so is milk*. *It cannot be associated with anything bad*.*”* (Interview-Elder-Endulen- Ngorongoro)

## Discussion

The findings of this study suggest that perceptions of zoonosis are still developing, as there is no agreed conceptualization. There is a widespread absence of the notion of a zoonotic illness and a disbelief that a zoonosis can be transmitted through consumption of animal products, in particular meat and milk, especially among the participants from the two eastern districts. Pastoralists in Ngorongoro were somehow more articulate about the existence of zoonosis than those from Kibaha and Bagamoyo, possibly implying differences in belief systems or awareness about illnesses across the different settings. This understanding has been influenced by people’s own local knowledge and by experiences about livestock and human illnesses, but also their interaction with medical and animal health services.

Knowledge of zoonoses in these communities, as framed in biomedicine, is at best still evolving and is confirmed by pastoralists’ frames of references of what constitutes an infectious disease caused by livestock pathogens crossing into the humans. Pastoralists participating in this study, no doubt believe that there are similarities between some syndromes that occur between animals and humans, but whether these constitute a zoonosis as defined and understood in biomedical sciences remains unclear. This calls for more research, and thereafter communication of findings back to the community. Findings from a previous study conducted in northern Tanzania showed that participants confused zoonotic conditions with other non-zoonotic ailments [8]. This suggests our results may demonstrate the likelihood of increased knowledge within the area. A number of reasons could be attributed to this improved knowledge. This includes the availability of tests especially for brucellosis in the local hospitals, but also research and community studies on zoonoses in the region [19,45].

Despite threats posed by zoonoses to human health, participants expressed little concern. To understand pastoralists’ indifference to animal diseases, especially as displayed by the Maasai, one cannot but embrace their conception of health, illness and causality, which is rooted in their cosmology. As Arhem [46] and Westerland [30] contends, Maasai do not conceptually distinguish between “supernatural” and “natural” illnesses and their concurrent treatments in herbals and ritual medicine, which derive their power from God [47]. Maasai’s knowledge on what causes disease is also different since they do not necessarily employ conventional understanding to categorize illness causative agents into viruses, bacteria, parasites and fungi [27]. Locally, studies about causative agents of tuberculosis among pastoralists and agro-pastoralists in northern Tanzania did not mention microbes as the suspected cause in humans [8,48–50]. The absence of local knowledge on microbial and parasitic causative agents of disease that can survive in both the humans and animals may explain the low understanding of zoonoses in this study including their unrelenting risky practices. This understanding is not limited to pastoralists in Tanzania alone; pastoralists elsewhere do not believe that milk consumption is a significant transmission pathway for pathogens as they too lack knowledge of contagious microbial agents [29].

The results further showed that pastoralists do not conceive the threats of zoonosis unless they have been able to link the biomedical realities, for example, from hospital or veterinary diagnosis with the visible or actual disease. The perceptions may also be muted by indigenous practices, which render the food safe, such as the use of herbs during cooking and eating of meat and milk. Furthermore, populations who experience high exposure levels over long periods, to pathogens in undercooked meat and milk may develop immunity over time. Risk or danger [expressed here in terms of fear] is actually expressed by the desire among pastoralists of the need for physical verification that the animal is in fact infected. It appears that perceived susceptibility to zoonoses becomes a reality only when they can visibly confirm the symptoms of a condition in the animal and the extent of decomposition in the carcass. This conviction calls for a culturally adapted but relevant communication, education and information strategy. Such a strategy can make an impact if it is rolled out over a period of time, and if it is a strategy engaging multiple stakeholders from the regional, district down to the village level.

The understanding of the possibility of an infectious disease being transmitted from animals to human vis-à-vis their known risky practices needs to be approached carefully. For example, previous studies on pastoralist local knowledge of disease transmission have demonstrated how the South Sudan Dinka cattle keepers who did not regard anthrax as transmittable to humans and will therefore dissect a dead carcass, cook and eat it [33]. This practice is done to all animals that die. All the social groups in our study affirmed this practice although the Maasai recognize human infection and a subsequent death will not always be caused by the infection in the animal, which is why they will also consume meat from an infected animal. This practice among the Maasai seems to be a long and ongoing one as it has been documented elsewhere [51].

To appreciate the local knowledge about known and propagated practices of the pastoralists that puts them at risk of acquiring infectious diseases, such as drinking of raw milk, and subsequent known alternatives, also needs further scrutiny. For instance, human subsistence of *Maasai* pastoralists has mostly been measured through milk availability [52]. The benefits of raw milk are undeniably both culturally and scientifically correct, but the presence of pathogens through unsafe milk preparation or from infected livestock renders it unsafe [53]. However, campaigns on preventing disease transmission such as boiling milk rarely consider these long known benefits of raw milk, which the pastoralists hold dearly and defend. Further, such solutions ignore the cultural and socio-political context upon which pastoralists live [54]. Put differently, if raw milk is considered as food [or a main staple food] by some groups of people, then milk pasteurization may imply going hungry. In his study about Maasai food as symbolism, Arhem [55] portrays how milk, like other foods, is as much culturally constructed as materially produced. Innovative alternatives to milk pasteurization may be needed while preserving its benefits as held in the local context. Although Maasai are changing their diet to consume more grain, milk will remain an important part [56]. This is likely to contribute to continued risk exposure by pastoralists even though they are becoming aware of zoonotic conditions and their transmission pathways. Interestingly, some studies do emphasize the benefits of raw milk in asthmatic persons and as an allergy prophylaxis [57][58]. Therefore, introducing solutions to raw milk in pastoral communities may need to consider such developments since changing behaviors in complex and marginal environments demand sustained efforts over time.

The consumption of undercooked meat and drinking raw blood reported by pastoralists show how just both cultural and social factors in environmental context considerably mediate the impact of infectious agents on humans. The social practice of eating undercooked meat and the drinking of raw blood from goats and cattle have the potential of causing a number of zoonoses. These include tuberculosis, anthrax, and brucellosis. However, these seemingly harmful practices are performed within an established system of life whose function goes beyond a mere consumption of these products in the manner that they do. They maintain order and social cohesion, instill bravery and help build respect between and among different age set groups [55,56]. A discrepancy between government control measures and local realities may hinder long-term community engagement and uptake of health officials’ directives. Although the regular consumption of blood has drastically waned due to, among others, reduction of livestock numbers, drinking raw milk is still part of their main diet. The negative attitude towards the local health officials can perhaps be viewed in part as a resistance to suppression of pastoralist’s way of life. From a different point of view, these actions may be viewed as the government’s way of utilizing disease and disease control as a political and economic vehicle through which tariffs and other taxes or sanction can be applied [59]. But the extent to whether pastoralists currently, will intentionally conduct harmful practices even after some have acquired modern medical and veterinary knowledge will demand further investigation.

The resistance towards associating tuberculosis among pastoralist in coastal districts of Tanzania could be because bovine tuberculosis [the zoonotic TB] was not commonly known about. What seems to be popular was the knowledge that the human form of TB was not transmitted through the consumption of raw milk from cattle. This could also be explained by some studies conducted on in pastoral communities in northern Tanzania, which showed the prevalence of bovine tuberculosis to be extremely low [60]. When put within the pastoralists’ social, economic and cultural context, the resistance could further be explained by the fact that milk is also an important source of income and nutrition and in the hands of the women. Associating it with TB would signal it was not fit and will hinder its sales.

There might be reasons to believe that Maasai pastoralists refused to accept diseases such as TB [especially human TB] because of the stigma it posed in some Maasai communities, where is sometimes associated with HIV/AIDS. A number of recent studies from similar groups and socio-ecological studies documented a gap in knowledge on the perceived causative agents of tuberculosis [48,49,61]. In their study about perceptions of tuberculosis among Maasai of Simanjiro, Haasnoot and colleagues [48] documented multiple reported causes of tuberculosis such as staying for long periods in strong sun, excessive exercise, smoking, promiscuity, breathing in dust and most reported that it was hereditary. It was also noted that God brought TB as a punishment to sinners. In light of this study future research on local knowledge about zoonotic TB should therefore strive to conceptually differentiate on the onset what version of TB people in that community recognize.

## Conclusion

The study has shown that the knowledge and perceptions about zoonoses by pastoralists, as understood in the biomedical field, is at best still evolving. Using their own indigenous knowledge frameworks pastoralists understand the possibility of a few infections that could cross over to humans from animals but harm from these are generally tolerated and are not considered as threats. It also showed that perceived risks of zoonoses were overshadowed by local knowledge in cultural practices and the value of animal source food. There are contradictions in perceptions and perhaps knowledge of zoonoses in these settings between local people and professionals. Therefore, to better understand perceptions about zoonoses it is equally pertinent to understand how pastoralists perceive animal diseases and meaning of illness and health management within their socio-cultural context. Evidence is needed to better understand the true burden and impact of zoonoses in these communities and to clearly categorize other risk factors if disease burden is confirmed. Once there is more clarity and common understanding on zoonoses, then interventions will be more effective and accepted. A synergy of scientific work around zoonoses and peoples’ social and cultural practices needs to be carefully tailored to allow for infusion of scientific understanding by pastoralists.

## Supporting information

S1 File Interview guide sample questions. (DOCX)
