# Supplementary material for: Exploring local knowledge and perceptions on zoonoses among pastoralists in northern and eastern Tanzania
Source: PLoS Negl Trop Dis. 2017 Feb 1;11(2):e0005345. doi: 10.1371/journal.pntd.0005345 (PMC5325590; doi:10.1371/journal.pntd.0005345)
Supplement: S1 File — (DOCX) [file pntd.0005345.s001.docx]

## S1 File. Interview Guide Sample questions

| **Domain** | **Topics and probes** |
| --- | --- |
| Warm up | 1. Can you tell me in your experience what are the most important issues facing pastoralists in your community? |
| Knowledge and Perceptions of diseases | 1. What are the diseases/illnesses with high impact on pastoral livelihoods including livestock?   *Initial Probes:*   - Name 5 common human diseases - Name 5 common livestock diseases - Rank each in the order of importance |
|  | 1. On the diseases mentioned above kindly explain the following:   *Initial probes*   - - Explain the symptoms of each   - Explain with examples how one can get it   - Explain with examples which period of the year do they occur   - Explain how you manage each disease: seeking care and treatment methods |
| Knowledge of diseases affecting animals and human [Zoonosis] | 1. What are the diseases that can affect both humans and animals?   *Initial Probes:*   - What is the local term for diseases affecting both animals and humans? - What are the diseases affecting both animals and humans?  1. Can you please explain to me how and why each of the disease you mention affects both humans and animals?   *Initial Probes:*   - - The etiology, source, transmission, symptoms   - Real experiences or incidences, past or existing, of encountering zoonosis and how these were addressed by different people in the community     - Who is responsible for identifying these symptoms and     - How is the management or treatment seeking behavior implemented once the symptoms are identified     - Health seeking pathways noting any differences between local to modern approaches     - Probe for reasons behind these treatment pathways *[Focus on specific diseases with detailed experienced cases]* |
| Population at risk | 1. Who are the people most at risk of acquiring zoonoses and why? |
| Local practices contributing to zoonoses | 1. What are the reasons/factors that can contribute to one acquiring zoonoses? Explain each answer with detailed examples?   Initial probes:   - Probe on social and behavoural activities - Probe around cultural, traditional responsibilities - Probe for risk activities around abattoirs and markets |
